# Medicinal Product Development and Regulatory Agilities Implemented During the Early Phases of the COVID-19 Pandemic: Experiences and Implications for the Future—An Industry View

**DOI:** 10.1007/s43441-023-00536-y

**Published:** 2023-06-02

**Authors:** Gaia Geraci, Janis Bernat, Céline Rodier, Virginia Acha, Jaqueline Acquah, Ginny Beakes-Read

**Affiliations:** 1 Clarivate, 70 St Mary Avenue, London, EC3A 8BE UK; 2 IFPMA, Chemin des Mines 9, 1202 Geneva, Switzerland; 3 Clarivate, Paris, France; 4 MSD, 120 Moorgate, London, EC2M 6UR UK; 5 Johnson & Johnson Middle East FZ-LLC, 3rd Floor Nestar Square, Airport City, Accra, Ghana; 6 Amgen, Inc., Global Regulatory and R&D Policy, 601 13th Street, NW, Suite 1100 North, Washington, DC, 20005 USA

**Keywords:** Regulatory, Agilities, COVID-19, Quality, Clinical trials, Regulators, Reliance

## Abstract

The COVID-19 pandemic caused considerable disruption to the development, regulatory evaluation, production, and distribution of medicinal products. Key healthcare stakeholders were under pressure to develop and review medicinal products to address the health emergency, while preserving the continuity of activities to ensure patient access to other medicinal products. In the light of this challenging situation, the National Regulatory Authorities (NRAs) and the biopharmaceutical industry applied and utilized product development and regulatory agilities to accelerate the development and authorization of safe, effective and quality COVID-19 vaccines and treatments as well as other non-COVID-19 medicinal products. On the basis of the literature review and primary research conducted, this review article gathered insights on experiences and challenges in the use of agilities related to regulatory assessment of initial marketing and post-approval change (PAC) applications, oversight of product manufacturing quality and supply chain continuity, and product development/clinical trial processes during the early phases of the COVID-19 pandemic. Agilities were thus implemented in an emergency context characterized by the lack of medicinal products to help tackle a disease that was devastating for the global public health. This review article concludes that useful lessons can be learned from these insights to improve product development practices and regulatory processes during both normal and health emergency times. Standard regulatory frameworks during normal times can be enhanced by leveraging digitalization, further simplifying and harmonizing requirements, and using reliance mechanisms which can help to increase efficiency in regulatory decision-making regarding medicinal products. During health emergencies, such as a pandemic, maximizing global coordination, collaboration, reliance, and harmonization of regulatory requirements and guidance are important to facilitate the rapid development and assessment of key medicinal products to address the health emergency.

**Supplementary Information:**

The online version contains supplementary material available at 10.1007/s43441-023-00536-y.

## Introduction

The COVID-19 pandemic brought new challenges, and exacerbated existing ones, for all stakeholders involved in the development, evaluation, production, and distribution of medicinal products. In an effort to overcome some of these challenges, the National Regulatory Authorities (NRAs) and the biopharmaceutical industry applied and utilized a variety of extraordinary product development and regulatory agilities to facilitate the rapid development, assessment and authorization of safe, effective and quality COVID-19 vaccines and treatments, as well as non-COVID-19 medicinal products.

This review article provides an overview of experiences and challenges in the use of agilities related to regulatory assessment of initial marketing and post-approval change (PAC) applications, oversight of manufacturing, quality and supply chain continuity, and product development/clinical trial processes, during the early phases of the COVID-19 pandemic. The insights focus primarily on global trends, but some regional experiences are also covered.

On the basis of the insights reported, this review article also offers lessons and recommendations to prepare for the future, whether in the normative (non-emergency) context or in case of a future health emergency, such as a pandemic. This review article offers an industry viewpoint. In the primary research phase only industry members were interviewed; no NRAs or patient advocacy groups were interviewed. These stakeholders may have different perspectives on the use, challenges and value of agilities in emergency and non-emergency situations and in the future, it will be important to also explore their views. Nonetheless, it is hoped that the insights, lessons and recommendations offered can facilitate informed discussions and planning among key stakeholders on how to improve the development and regulatory processes of medicinal products in both emergency and non-emergency situations.

## Materials and Methods

This review article offers insights gathered via literature review and primary research. A total of 36 documents (including documents published between June 2019 and December 2021 as well as unpublished internal documents/analyses), selected by the International Federation of Pharmaceutical Manufacturers & Associations (IFPMA), were reviewed; these included reports, publications, and surveys (listed in the Appendix) related to product development and regulatory agilities that emerged during the pandemic, with global coverage. To supplement the results from the literature, four one-hour interviews were conducted by Clarivate in January 2021 with IFPMA members including regional trade associations and manufacturers, who shared experiences related to the following regions or countries: Africa, Asia, the United States, Latin America. The interviewees were selected on the basis of their professional roles and knowledge related to the implementation of product development and regulatory agilities in their regions or countries.

The analyzed documents were deemed to cover enough insights from the European Union (EU) as well as the United Kingdom (UK); therefore, no interviews were conducted for the EU and the UK. The analysis was complemented with the experience shared during four meetings by the IFPMA Steering Group, composed of 10 IFPMA members.

## Results

Reported experiences related to agilities were analyzed and grouped into three categories (regulatory assessment of initial marketing and PACs applications; oversight of manufacturing quality and supply chain continuity; product development/clinical trials procedures) as further explored below.

### Regulatory Assessment of Initial Marketing and PACs Applications

Agilities related to certain regulatory processes included processes for submission and assessment of applications for marketing authorization and PACs.

#### Digitalization of Working Practices

Restrictions of movement to limit the spread of the virus led to increased digitalization of working practices. Use of digital methods and electronic documents, such as e-submission of applications, e-signatures, and the e-Certificate of Pharmaceutical Product (eCPP), were successfully leveraged by the NRAs to increase efficiency and facilitate continuity of activities [1–4]. The NRA staff often had to remain at home and rely on their devices and internet connection to support on-line document exchange and teleconferencing. In some low-income countries however, regulatory processes were sometimes interrupted due to a lack of reliable and secure infrastructures such as online platforms or email systems [5].

#### Risk-Based Approaches in Decision-Making

To enable rapid authorization of quality medicinal products, in particular COVID-19 vaccines, many NRAs successfully employed ad-hoc risk-based approaches in their decision-making processes, which took into account the high public health risk of a pandemic without medicinal products to help to tackle it. These approaches were still employed respecting regulatory standards. It should be stressed that a benefit/risk approach is inherent in all regulatory decision-making processes, which are thus all risk-based. The terminology “ad-hoc” is used to stress that these approaches were taken within the COVID-19 pandemic emergency context. Exceptional authorizations were for instance granted to products as soon as enough data became available to support the probability of public health benefit, and dossiers were completed with the remaining required data to demonstrate public health benefit at a later stage. Key examples of ad-hoc risk-based approaches include the use of rolling submissions and reviews, enabling data to be assessed as soon as it became available, and the use of other approaches which already existed, such as reviews under Article 5(3) by the EMA [6, 7] and exceptional authorizations such as conditional marketing authorization (CMA) by the EMA and emergency use authorization (EUA) by the US FDA [8–10]. These approaches were not, however, equally applied by the NRAs around the globe. In some regions, for example, rolling reviews and expedited authorizations were mostly used by authorities with more resources and not by less mature NRAs (interview insight). Even though not an authorization per se, the emergency use listing (EUL) by the WHO is also an example of an ad-hoc risk-based procedure implemented by an international organization aimed at facilitating national authorizations [11].

#### Post-Approval Changes (PACs)

Agilities were also allowed for PACs such as approvals in the absence of complete data (with certain data provided at a later date), expedited procedures for extensions of indications for approved medicines being repurposed for COVID-19, and postponements of certain PACs [3, 8, 9, 12].

Regulatory process agilities were sometimes also applied to non-COVID-19 related products. In the United Kingdom, for instance, expedited assessment of variations and initial applications for products which could facilitate continuity of the supply chain were allowed [13].

#### Increased Collaboration

The role of the International Coalition of Medicines Regulatory Authorities (ICMRA) as a forum to support strategic coordination and international cooperation among many NRAs was central during the pandemic. The ICMRA activities aimed to expedite and streamline the development and authorization of COVID-19 medicinal products and increase efficiency in regulatory decision-making [14]. In particular, examples of the achievements of the ICMRA COVID-19 Working Group range from a statement on clinical trials to a joint statement with the WHO to help healthcare professionals increase trust in COVID-19 vaccines and answer questions from patients about the vaccines [15].

Some NRAs sought to increase efficiency in their assessment and authorization of COVID-19 products by engaging with other NRAs in early scientific dialogue and collaborative assessments, such as via the EMA’s OPEN pilot project. This pilot project was considered to be an important collaboration opportunity by the parties involved, and allowed the WHO and the NRAs from outside the EU to join the EMA in scientific evaluations and share their scientific expertise [10].

#### Reliance

The NRAs of various maturity levels implemented ad-hoc risk-based approaches, such as the use of reliance, to increase efficiency of regulatory review and to save stretched regulatory resources. Through reliance, the NRAs take into consideration decisions and assessments of other NRAs in their decision-making processes. This allows the NRAs to leverage the outputs of other NRAs and dedicate greater focus at the national level on other value-added regulatory activities [16]. According to a survey among 11 NRAs on agilities used to increase manufacturing capacity of COVID-19 products (including the US FDA, EMA, PMDA, and MHRA), conducted by the ICMRA, when asked which mechanisms the NRAs offered to expedite assessment of chemistry, manufacturing and controls (CMC) changes, 100% of these 11 NRAs said reliance on assessments by other agencies or participation in joint assessment programs and 64% said full or partial reliance on assessment reports of authorities from other regions. When asked about regulatory tools available for facility assessment in lieu of inspection, 91% of the NRAs said review of inspection reports by other agencies via a Mutual Recognition Agreement or Confidentiality Agreements [12, 17].

Moreover, some countries successfully relied on recommendations by the WHO for emergency use listing (EUL) for some COVID-19 vaccines. Notably, the WHO EUL procedure resulted in regulatory authorizations in over 100 low- and middle-income countries (LMICs), many within just 15 days of issuing an EUL, offering a very successful example of the use of reliance [18].

Despite these success stories, a key challenge identified by the industry was the considerable heterogeneity of the global regulatory landscape and lack of convergence and harmonization in regulatory requirements with respect to evidence, review and authorization processes [19, 20].

#### Labeling and Packaging

Agilities were often applied to labeling and packaging requirements. In particular, exemptions and waivers for labeling proved helpful for vaccine rollout [10]. In the EU, agilities such as e-labeling and single-language label formats for COVID-19 products were considered very beneficial by the industry [21].

### Oversight of Manufacturing Quality and Supply Chain Continuity

#### Digitalization of Working Practices

Agilities to ensure continuity of manufacturing quality assurance and supply chain integrity included extensive use of digital tools. Examples included use of e-signatures, e-documents such as electronic Good Manufacturing Practice (eGMP) certificates, and eCPP [2, 8]. Overall, working practices were increasingly virtualized to facilitate continuity of activities. For instance, the remote working of “qualified persons” and “responsible persons” was highly valued in Europe by the industry [21].

#### Remote and Hybrid Inspections

To limit disease transmission and respect travel restrictions, GMP inspections by the NRAs (both domestic and foreign inspections) were at times held remotely or using a hybrid approach. Remote and hybrid inspections were enabled by digital tools, obviating the need for a site visit by the inspectorate as appropriate. According to the ICMRA survey previously mentioned, when asked about the regulatory tools available for facility assessment in lieu of inspection, 100% of the 11 NRAs surveyed mentioned desk-based reviews of documents requested from the facility as well as remote interactive assessment or distant assessments [12, 17]. Another survey of the European Federation of Pharmaceutical Industries and Associations (EFPIA) in 2020 showed that less than 25% of foreign inspections were conducted with on‐site presence [22].

Despite the obvious benefits of remote and hybrid inspections, industry members experienced several challenges in this area. Insights from interviews indicated that industry considered on-site inspections, at times, to be more efficient than remote inspections. These remote inspections were often perceived by the industry to be longer and more resource-intensive, as they required additional planning and preparation compared with on-site inspections [23–25].

#### Extraordinary Procedures and Approaches

Various agilities were adopted by the NRAs to speed up manufacturing and to implement appropriate controls to enable production of COVID-19 medicinal products with acceptable quality standards given the public health emergency. For GMP compliance certifications, some NRAs allowed the use of extraordinary procedures, such as extensions of GMP licenses, extensions to fulfill requirements, and applied ad-hoc approaches to define which GMP activities could be delayed or modified [1, 21].

#### PACs

The accelerated product development and manufacturing processes to accommodate supply led to the need for a high number of PACs. To expedite CMC changes, NRAs allowed agilities such as concurrent process validation, approval of PACs in the absence of complete data (with data to be provided at a later stage) and derogations from labeling requirements [12]. In Africa for example, to ensure supply continuity, renewals and PACs were prioritized over non-COVID new drug applications [26]. In several Asian countries grace periods for the implementation of PACs were granted [27]. Additionally, Post-Approval Change Management Protocols (PACMPs), a regulatory tool proposed by the International Council for Harmonization of Technical Requirements for Pharmaceuticals for Human Use (ICH) guideline Q12, were considered by both NRAs and the industry as helpful to support and expedite PACs [17].

While agilities related to PACs were beneficial, the lack of global, harmonized requirements represented a considerable hurdle for the industry [17]; for instance, NRAs often have divergent expectations for the same PACs [28].

Post-approval site transfers of a product to additional manufacturing facilities, which were key to increase manufacturing capacity, also created challenges. At times, all data from process validation, real-time stability studies, and comparability testing (all resource-intensive processes) were required by the NRAs, and many NRAs were unfamiliar with science- and ad-hoc risk-based approaches which other NRAs were employing during the pandemic [12]. Moreover, accelerated stability testing, modelling, extrapolation, and/or prior knowledge as surrogates for real-time testing are not allowed by many NRAs [28].

#### Reliance

As mentioned before, in order to expedite assessment of CMC changes, some NRAs allowed full or partial reliance on assessment reports of authorities from other regions, which was considered key to increase manufacturing capacity for COVID-19 products from an industry perspective, as well as the review of inspection reports by other agencies if mutual recognition agreements (MRAs) or confidentiality agreements were in place [12, 17]. The NRAs increasingly granted waivers on good manufacturing practice (GMP) and good distribution practice (GDP) inspections for sites in a country where a memorandum of understanding (MoU) was established or when the inspectorate was a member of the Pharmaceutical Inspection Co-operation Scheme (PIC/S), provided that the required tools (such as on-site, virtual, paper-based, reliance on prior knowledge) were available [22].

### Product Development/Clinical Trials Procedures

#### Digitalization of Working Practices

Leveraging virtual tools helped promote continuity of clinical trial activity during the pandemic. Several NRAs and applicants increasingly exchanged information electronically and sponsors used for instance remote informed consent and e-signatures [8]. However, at times, the lack of appropriate technology infrastructure represented a barrier to virtualization of clinical trials working practices. For example in Africa, in the absence of dedicated e-portals, physical copies were requested for clinical trial applications (CTAs) [26]. In Japan, one interviewee noted how the use of local languages in clinical trial databases reduced accessibility [29].

#### Innovative Approaches in Design and Conduct of Clinical Trials

Innovative approaches in the design and execution of clinical trials were implemented to minimize the risk of disease transmission among participants and support study continuity. Clinical trials increasingly adopted some level of decentralization, allowing, for instance, remote monitoring of trials and direct-to-patient delivery of the investigational medicinal product at home [8]. The use of remote Source Data Verification (rSDV), was permitted by several NRAs such as the US FDA, EMA and MHRA, under certain conditions [21, 30–32].

#### Extraordinary Procedures

Other important agilities included the prioritization of reviews and authorization of studies for research related to COVID-19 and justified protocol deviations without the need to notify agencies (although sponsors still had to maintain relevant documentation to allow for assessment of trials) [8].

Burdensome administrative regulatory requirements as well as variations in requirements for clinical trial processes between countries were identified as challenges by industry members. A survey conducted by the EFPIA, Vaccines Europe, and Medicines for Europe in the EU, showed that the benefit of agilities for clinical research provided by EU harmonized guidance (such as for amendments, direct-to-patient delivery and remote assessment) was still limited by the national requirements of member states [20].

#### Increased Collaboration

To further support research and development, NRAs published guidelines and their interactions and communications with the industry became more frequent and scheduled whenever required during the pandemic [12]. For instance, the US FDA offered guidance to the industry for the development of COVID-19 treatments, including how to engage with the agency and speed up clinical trial initiation [33]. In the EU, the EMA offered sponsors guidance to facilitate protocol amendments and initiate new trials for COVID-19 treatments [21], as well as rapid scientific advice and access to a dedicated early point of contact at the EMA [20]. The UK MHRA also provided guidance covering how to manage clinical trials during COVID-19 [13].

### Additional Insights From Interviews

To complement the insights gathered via literature review, four members of IFPMA were interviewed.

When asked what regulatory agilities were most valuable during the pandemic in their regions and should be included in an international pandemic-preparedness toolbox, the most cited agilities were accelerated processes (i.e., expedited authorization) and reliance mechanisms (by 3 out of 4 interviewees), followed by digitalization and remote working (2 out of 4).

As key enablers for regulatory agilities in times of pandemic, interviewees identified digitalization (3 out of 4), political will (2 out of 4), and clear criteria for prioritization of products to benefit from agilities (2 out of 4).

**Fig. 1:**
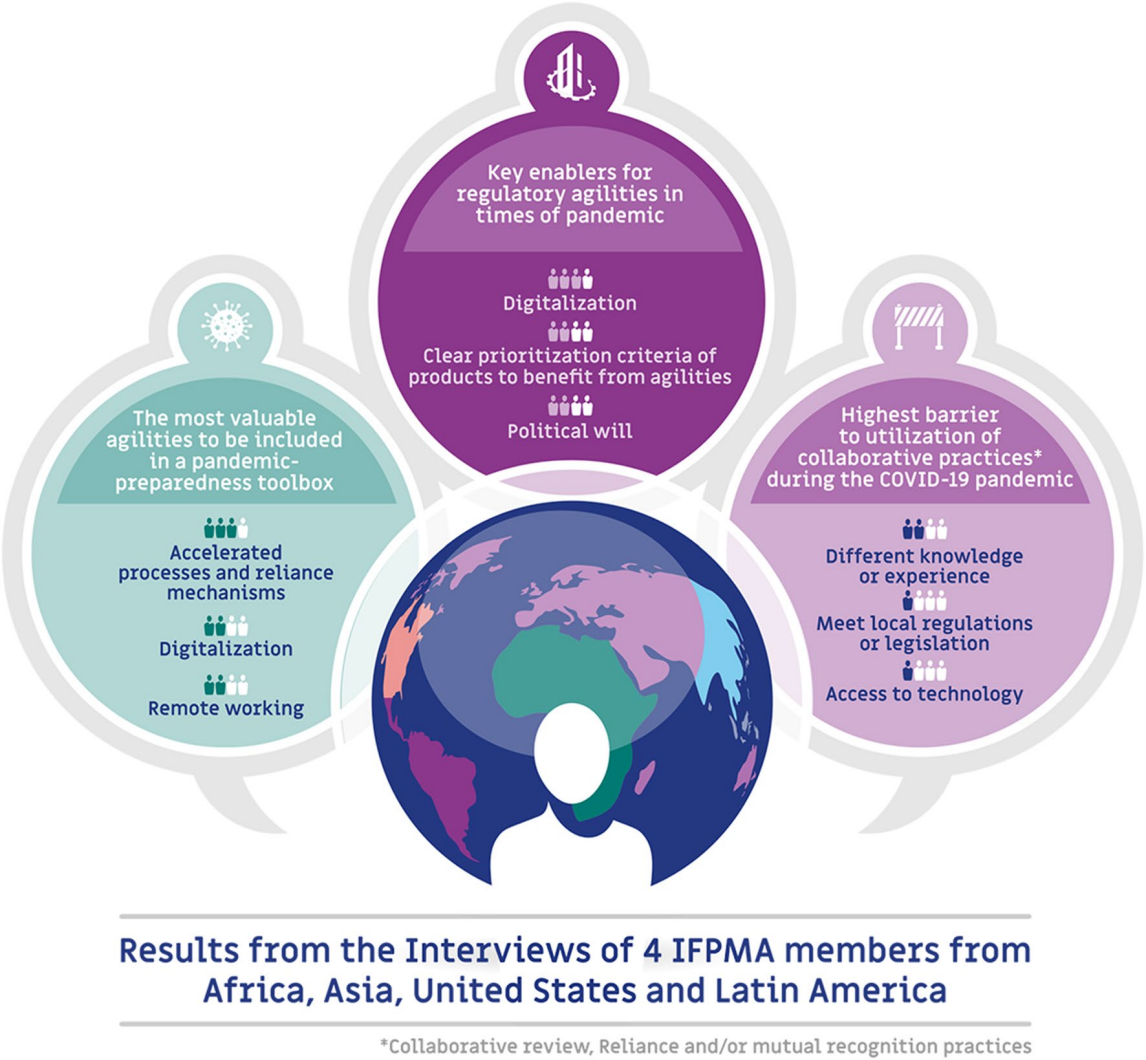
Results from the four interviews

In terms of the greatest barriers to utilization of collaborative review, reliance and/or mutual recognition practices in the regions in the context of COVID-19 pandemic, interviewees identified different knowledge or experience (2 out of 4), the need to meet local regulations or legislation (1 out of 4) and access to technology (1 out of 4) (Fig. 1).

As previously mentioned, these interviews only reflect the views of industry members interviewed; the NRAs and patient groups were not interviewed.

## Discussion

The data analyzed showed that the COVID-19 pandemic led to both the emergence of new practices, enhanced utilization of already existing emergency procedures and authorizations, and the acceleration of existing trends in regulatory processes such as the digitalization of ways of working and the use of rolling reviews.

Overall, agilities have proven beneficial in supporting continuity of regulatory activities and fast authorizations of much-needed quality medicinal products. Experience in the use of agilities offers useful insights on what has worked well and should continue to be implemented, what could be improved, and what hurdles should be addressed to enhance standard normative regulatory processes and preparedness for future pandemics. It should be noted that addressing the COVID-19 emergency and rapidly implementing agilities were very resource-intensive activities, for both the industry and the NRAs, especially in the early months of the pandemic. Carrying out similar activities and processes at a similar pace would be unsustainable in the long term, especially in non-emergency settings. The lessons and recommendations outlined for non-emergency settings, were selected taking into account these sustainability concerns. Furthermore, global lessons and recommendations offered in this review article are not tailored to specific countries or NRAs. For this reason, some recommendations may be less feasible for the NRAs with lower capacity and resources.

### Enhancing the Standard Normative Process

To improve standard normative processes, further digitalization of working practices should be encouraged. The use of digital methods such as e-signatures, e-documents, and virtual meetings can accelerate processes and increase efficiency. E-labeling, for instance, should be adopted when possible as it can improve speedy deployment of information, improve transparency and enhance patient safety by easily providing updated product information and facilitating information-sharing among stakeholders [34]. Moreover, digitalization may also support sustainability: e-signatures and e-labeling remove the need for printing, whilst e-submissions, virtual meetings, and virtual inspections can reduce travel, lowering environmental pollution. Digitalization may also have other positive effects such as facilitating document tracking and accelerating processes, as well as enabling reliance by easing information sharing and transparency. Promoting access to reliable digital infrastructure, by investing appropriate resources into this sector, should be a priority for regions with sub-optimal digital infrastructure to promote continuity and efficiency in regulatory activities. Whilst it is realistic to expect a general trend towards increased digitalization of working practices for the NRAs of advanced economies and with good level of resources, digitalization of working practices may happen at a much slower pace in regions where building reliable digital infrastructure poses significant costs for stakeholders and where more time may be needed to adapt to new ways of working.

Greater collaboration among all NRAs should continue to be fostered to create synergies. Data-sharing and transparency among the NRAs should be encouraged (while protecting confidential data from public disclosure and without undermining intellectual property), including sharing of full scientific assessment and inspection reports (for those NRAs who do not do so already). This would ease and support reliance-based and other facilitated regulatory pathways to the benefit of the industry, the NRAs, and patients. NRAs’ reliance on other NRAs’ regulatory decisions and GMP inspections should be encouraged to avoid duplication of effort and to promote trust among all NRAs. In order to facilitate the use of reliance, the WHO and international cooperative groups such as the ICMRA should continue to offer guidance and best practices on the use of reliance. All NRAs should also embrace the WHO Good Reliance Practices (GReP). The GReP outlines how reliance practices should always respect the overarching principles of fully-informed decision-making sovereignty, transparency, consistency and respect of local regulations or legislation, including those with protection of confidential data from public disclosure [35].

Ad-hoc risk-based approaches to decision-making, outside of the standard regulatory processes/pathways, should be used, by definition, exclusively on an ad-hoc basis. Ad-hoc risk-based approaches, such as rolling reviews and emergency use authorizations, should be applied in the context of particular urgency, for instance to accelerate the assessment of products aimed at tackling high unmet needs. This is already the case for some NRAs and hopefully the recent pandemic experience will encourage more NRAs to utilize ad-hoc risk-based approaches when needed. These approaches considerably impacted on workloads, as well as human and financial resources, thus different NRAs should consider assessing their usefulness and applicability as needed depending on their resources and specific circumstances.

Alternative approaches to GMP inspections could also be used on an ad-hoc basis; rather than fully replacing on-site inspections, remote and hybrid inspections should be considered and utilized as additional tools that the NRAs can leverage, depending on the context.

Guidance from international bodies should be embraced to improve, and where possible simplify and harmonize, regulatory requirements and practices globally in non-emergency times. Examples may include principles of the WHO Good Regulatory Practice (GRP) and the GRelP or integrating guidance from the ICH, such as ICH Q12, to facilitate PACs management. Special and targeted support should be given to countries with limited resources and less mature regulatory systems to help build efficient and effective regulatory systems and to implement international guidelines. Embracing guidance from international bodies would also help all NRAs to ensure a common degree of knowledge and understanding of suggested practices, which would enable further collaboration and reliance-based mechanisms.

Innovative approaches to the design and execution of clinical trials–such as decentralized clinical trials and the use of technologies to support them–should be incentivized to improve efficiency and speed up clinical development. By bringing clinical trials closer to patients and reducing the burden on patients (as well as on investigators), decentralized and hybrid clinical trials can facilitate patient participation and help to promote diversity and inclusion in clinical research. In the future, it will be important to assess the benefits derived from the implementation of decentralized clinical trials to gather evidence to further improve and encourage the use of such innovative approaches to clinical research.

More generally, in non-emergency times, all stakeholders should strive to enhance product development and regulatory practices in an iterative and continuous process of adaptation, aiming to improve efficiency and effectiveness. Innovative and inclusive clinical research should be encouraged, as well as building or improving local production capabilities, ultimately supporting a diverse and resilient global supply chain and accelerating access to medicinal products of international quality.

### Next Pandemic Preparedness

To better respond to future pandemics, global coordination among all NRAs should be maximized and clear convergence in the emergency response should be promoted. In order to achieve this, both the WHO and the ICMRA should continue to play a key role in facilitating alignment among the NRAs and other stakeholders.

Collaboration among NRAs should increase in emergency situations, for instance by joining collaborative reviews and joint assessments. Sharing of data among the NRAs should be encouraged and reliance maximized. This is true for all NRAs, regardless of their level of maturity, but maximizing reliance becomes truly crucial in emergency times for less mature NRAs, allowing them to rely on assessments and reports of the NRAs with greater capacities. Recognizing that all NRAs are resource stretched during emergency times (as shown by the COVID-19 experience), by using reliance, all NRAs can save precious resources and re-allocate them to other important local matters.

To accelerate regulatory processes and avoid impasses, the development of regulatory frameworks for emergency circumstances, when they do not already exist, should be strongly encouraged. Such frameworks should be ready-to-use and include ad-hoc risk-based approaches for pandemic contexts, such as wider and novel use of accelerated submissions, rolling reviews and exceptional authorizations. As shown, ad-hoc risk-based approaches, in the context of the COVID-19 pandemic, proved key to enable accelerated development and authorization of efficacious medicinal products, whilst respecting quality and safety within the pandemic context. To develop optimal emergency frameworks, input from various stakeholders would be needed to ensure that the needs and concerns of different parties involved, such as the NRAs, the industry, patient groups and civil society, are taken into consideration.

Fast and increased communication among the NRAs and sponsors should also be encouraged to promote swift alignment on regulatory agilities and requirements. Surely, this communication must be well-documented and appropriate, respecting the boundaries between the NRAs and the sponsors, who need to comply with NRAs’ rules and regulations. Increased communication and alignment among the NRAs should also be encouraged as it can help the NRAs to improve the management of health emergencies and share best practices for decision-making. To ensure rapid communication among the NRAs and between the NRAs and sponsors, specific emergency points of contacts could be established where they do not exist already.

Requirements related to regulatory assessment of initial marketing and PACs applications, oversight of manufacturing quality and supply chain continuity, and product development/clinical trial processes should be streamlined as much as possible and harmonized globally to accelerate product development and assessment and to ensure better predictability and alignment between stakeholders. Harmonization and clarity on the agilities suggested for use during a pandemic would also be an enabler for reliance, avoiding delays due to unfamiliarity with alternative tools and procedures. PACs, for example, would highly benefit from more streamlined and harmonized frameworks, given the high number of PACs to be addressed during a health emergency. Prioritizing changes based on the health emergency, granting waivers, extensions, and aligning on a unified set of queries for each PAC become critical in such circumstances. It is also recommended to streamline labeling and packaging requirements, allowing for derogations when appropriate (as a result of CMC changes for instance) and the movement of a product between countries to meet emerging needs in a pandemic setting. Moreover, the use of e-labeling should be maximized to support resilience of the global supply chain and accelerate processes. It should be noted however, that streamlining and harmonizing regulatory requirements globally, even in case of a health emergency, will not be an easy endeavor, because countries have different local regulations and legislation. In order to facilitate this, the NRAs should engage in discussions, ideally through global forums such as the WHO and the ICMRA, and agree on at least some basic harmonized, or aligned, requirements for key areas (like review of dossiers or PACs) to follow during emergencies.

To expedite quality assurance processes, ad-hoc risk-based approaches to GMP certification and real-time data review can be particularly helpful. Depending on the context, remote or hybrid GMP inspections should be leveraged to accelerate processes and reliance on inspection reports of other NRAs, when appropriate, should be maximized to enhance efficiency.

To support and accelerate clinical research, the NRAs should provide guidance related to clinical trial conduct and evidence generation as early as possible and maintain an open channel of communication with sponsors. Alternative clinical trial designs and means of execution, which can expedite research, should be encouraged. Decentralized and hybrid clinical trials and the use of digital tools should be leveraged to accelerate product development, as they can facilitate trial recruitment, diminish the need for both trial participants and personnel to travel to sites, and contribute to ensure diversity in clinical trials. Agilities such as accelerated, including joint, assessment of CTAs, and use of innovative approaches such as alternative methods for participant consent, drug delivery, and remote SDV should also be considered as options to support medicine development. To ensure proper use and implementation of product development agilities, the NRAs should provide guidance to the industry to facilitate robust evidence generation to support product authorizations and approvals (as it often was the case during the COVID-19 pandemic). Partnerships and communication among these two key stakeholders should thus increase in emergency times for the benefit of the community, still preserving the roles of and boundaries among the NRAs and the industry, who needs to comply with NRAs’ rules and regulations.

In emergency settings, while prioritization of efforts will of course be directed to address the emergency, plans should be in place to ensure, when possible, continuity of non-pandemic related regulatory activities. This can help to minimize backlogs of activities or severe delays. This is of course particularly challenging to implement during an emergency when human and financial resources are stretched; having surge capacity proved difficult during the COVID-19 pandemic not only for organizations such as the WHO and the NRAs with lower resources but also for highly-resourced NRAs. A starting point might be agreeing on a list of non-pandemic related regulatory activities which should ideally continue, even if at a necessary slower pace. The stakeholder community should come together to explore ideas to address this challenge.

To ensure that key agilities are used during a pandemic in as many jurisdictions as possible, it is important that all stakeholders, including decision-makers, are aware of the benefits and challenges of regulatory agilities to secure the political will to implement them. As new agilities are likely to emerge, from future pandemics or other public health emergencies, all stakeholders should be invited to systematically share their knowledge and experience on the effectiveness and challenges of agilities to enable fully-informed discussions that will continue to strengthen global pandemic preparedness.

Moreover, in light of the successful development and commercialization of safe and effective COVID-19 medicines, it seems reasonable to think that during future health emergencies stakeholders may be more confidently applying agilities to accelerate the development and assessment of medicinal products without compromising safety and effectiveness in the context of the emergency. Even though patient access to COVID-19 medicinal products was not the focus of this review article, it seems appropriate to mention that for many patients, accelerated product development, assessments, and authorizations resulted in rapid access to much needed medicinal products. However, in many parts of the world such as low-income countries, use of agilities did not translate into rapid patient access due to various challenges (not explored in this review article) following product development and authorizations. This inequity in patient access should be addressed before the next public health emergency.

Overall, when responding to the next health emergency, global coordination, collaboration, reliance-based regulatory pathways, and harmonization of regulatory requirements and guidance should be maximized to facilitate the rapid development, assessment, authorization, distribution, and availability of medicinal products to address the emergency, while securing patient access to other medicinal products.

The recommendations offered in this review article aim to support future discussions with the wider stakeholder community on possible ways to enhance medicines development and regulatory processes in both emergency and non-emergency contexts. The recommendations offered should not be perceived as definitive. The co-authors believe that various stakeholders including the industry, the NRAs and patient groups should come together to collectively discuss the lessons learned from the use of agilities and best ways to implement the knowledge acquired moving forward in both an emergency and non-emergency context.

## Limitations

This review article does not aim to provide an exhaustive overview of all agilities used during the COVID-19 pandemic or to provide the views of the NRAs or patient advocacy groups regarding these agilities and their use during the COVID-19 pandemic. Reported trends and experiences related to the use of agilities may not apply to all countries, as some generalizations have been made. No systematic literature review was conducted for this study. Results are based on data contained in the selected materials reviewed (Appendix), as well as insights shared by interviewees. For these reasons, the insights on agilities and the related analysis offered by this review article may not be fully objective or comprehensive. Nevertheless, the information presented in this review article offers valuable insights on agilities implemented during the COVID-19 pandemic and lessons learned from this experience.

### Relevance for Policy Discussion

The results of this review article offer useful insights to facilitate informed policy discussions of global, regional, and national stakeholders on how to potentially improve product development and regulatory procedures during health emergencies such as pandemics and in standard times moving forward. Policies supporting the use of agilities related to the regulatory assessment of initial marketing and PACs applications, oversight of manufacturing quality and supply chain continuity, and product development/clinical trials procedures may be critical for enhancing standard regulatory frameworks as well as maximizing pandemic preparedness.

### Implications for Future Research

Future studies could assess the efficiency and effectiveness of different agilities, which could help to identify the most useful agilities to prioritize for future use.

More clarity on the perceptions of the NRAs and patient advocacy groups on the use of agilities would also be beneficial. Surveying the NRAs in different regions could help to better understand shared views as well as specific needs and challenges of the NRAs. Ultimately, this would help define which agilities should be part of a global pandemic preparedness toolkit and which ones should be adapted to specific regional use.

## Conclusions

Implementing product development and regulatory agilities during the COVID-19 pandemic played a key role in enabling accelerated research and development of medicinal products and in ensuring supply continuity of quality COVID-19 vaccines and treatments. Several lessons can be drawn, from the use of these agilities and related challenges, to enhance standard regulatory frameworks and preparation for future health emergencies, such as pandemics.

Leveraging electronic documents and digital methods proved critical in ensuring continuity of regulatory activity and accelerating clinical research throughout the COVID-19 pandemic. Increased digitalization can be further encouraged in standard regulatory processes to: improve efficiency, transparency, enable reliance, and advance clinical research, for instance through decentralized and hybrid clinical trials. The implementation of context-based agilities for decision-making processes, such as rolling reviews, successfully shortened timelines during the COVID-19 pandemic and can be considered for wider use in non-emergency times to accelerate product development and regulatory assessments of much needed medicinal products. Overall, the standard regulatory framework can be further strengthened by simplifying, harmonizing, and using reliance-based mechanisms to increase efficiency and to accelerate product development and assessment of all medicinal products.

The COVID-19 pandemic has demonstrated the importance of global cooperation in addressing health emergencies as well as the positive impact of agilities in accelerating regulatory and clinical research processes whilst maintaining context-appropriate quality, efficacy, and safety standards of medicines. In case of future pandemics, global collaboration, data sharing, transparency, reliance and harmonization of regulatory requirements and guidance should be maximized to facilitate the rapid development and assessment of medicinal products to address the health emergency.

## Supplementary Information

Below is the link to the electronic supplementary material.

Supplementary file1 (DOCX 33 KB)
