# Supplementary material for: Medicinal Product Development and Regulatory Agilities Implemented During the Early Phases of the COVID-19 Pandemic: Experiences and Implications for the Future—An Industry View
Source: Ther Innov Regul Sci. 2023 Jun 2;57(5):940–51. doi: 10.1007/s43441-023-00536-y (PMC10237066; doi:10.1007/s43441-023-00536-y)
Supplement: Supplementary file 1 — Supplementary file1 (DOCX 33 KB) [file 43441_2023_536_MOESM1_ESM.docx]

**Appendix**

List of the 36 documents (including documents published between June 2019 and December 2021, at time of the analysis, as well as unpublished internal documents/analyses), selected by the International Federation of Pharmaceutical Manufacturers & Associations (IFPMA) for the secondary research.

**Published sources:**

1. European Federation of Pharmaceutical Industries and Associations (EFPIA). *Annual Regulatory GMP/GDP Inspection Survey 2020 Data* 2021. <https://www.efpia.eu/media/602634/efpia-2020-reg-inspection-survey_v1a.pdf>
2. International Federation of Pharmaceutical Manufacturers & Associations (IFPMA). *Biopharmaceutical Industry Joint Statement on ICMRA workshop on enabling manufacturing capacity in the COVID-19 pandemic*. 2021. <https://www.ifpma.org/resource-centre/biopharmaceutical-industry-statement-on-icmra-workshop-on-enabling-manufacturing-capacity-in-the-covid-19-pandemic/>
3. International Federation of Pharmaceutical Manufacturers & Associations (IFPMA). *Considerations for effective regulatory reliance- an industry perspective*. 2019. <https://www.ifpma.org/wp-content/uploads/2019/06/IFPMA-Position-Paper-Regulatory-Reliance.pdf>
4. Food and Drug Administration (FDA). *Coronavirus Treatment Acceleration Program (CTAP)*. 2021. <https://www.fda.gov/drugs/coronavirus-covid-19-drugs/coronavirus-treatment-acceleration-program-ctap>
5. European Medicines Association (EMA). *COVID-19: how EMA fast-tracks development support and approval of medicines and vaccines*. 2020. <https://www.ema.europa.eu/en/news/covid-19-how-ema-fast-tracks-development-support-approval-medicines-vaccines>
6. International Federation of Pharmaceutical Manufacturers & Associations (IFPMA). *Diversity and Inclusion in Clinical Trials: Bioethical Perspective and Principles*. 2022. <https://www.ifpma.org/wp-content/uploads/2022/05/IFPMA-Global-Clinical-Trial-Diversity-Principles_May-19-2022-.pdf>
7. Fujiwara Y. *For Your Access to Japanese Clinical Trial/Clinical Research Information* (PMDA). 2020. <https://www.pmda.go.jp/english/int-activities/0006.pdf>
8. Rönninger S, Kurz A, Raya F. GMP/GDP Inspections: Challenges and Opportunities from COVID-19. *Pharmaceutical Technology*. 2021;45(11)
9. Mwangi J. *ICDRA Workshop 5: Building regulatory agility for local production to combat pandemics: An industry perspective*. IFMPA. 2021. https://cdn.who.int/media/docs/default-source/medicines/regulatory-updates/virtual-icdra-2021/icdra_session5_local-production_3_ifpma.pdf?sfvrsn=7db30194_7
10. International Conference of Drug Regulatory Authorities (ICDRA). *ICDRA Workshop 7: Good Regulatory Practices; Good Reliance Practices*. 2021. <https://cdn.who.int/media/docs/default-source/medicines/regulatory-updates/virtual-icdra-2021/extra-icdra_workshop-7_v3.pdf?sfvrsn=5e8bb0fd_5>
11. International Coalition of Medicines Regulatory Authorities (ICMRA). *ICMRA Statement on Pre-Requisites for Regulatory Flexibility in Pharmaceutical Manufacturing Change Management*. 2021. <https://www.icmra.info/drupal/covid-19/icmra_statement_on_flexibility_in_manufacturing_change_management>
12. International Coalition of Medicines Regulatory Authorities (ICMRA). *ICMRA-Industry Virtual Workshop on Enabling Manufacturing Capacity in the COVID-19 Pandemic*. 2021. <https://www.icmra.info/drupal/sites/default/files/2021-10/covid-19_manufacturing_capacity_ws_report.pdf>
13. International Federation of Pharmaceutical Manufacturers & Associations (IFPMA). *IFPMA Points to Consider for Virtual Inspections* 2021. <https://www.ifpma.org/resource-centre/ifpma-points-to-consider-for-virtual-gmp-inspections-an-industry-perspective/>
14. The International Federation of Pharmaceutical Manufacturers & Associations (IFPMA). *Improving Patient Safety and Health Systems Resilience Through the Use of Electronic Labeling*. 2022. <https://www.ifpma.org/wp-content/uploads/2022/02/IFPMA_Position-paper_Electronic-labeling.pdf>
15. Cooke E. *An overview of emergency use authorizations and similar authorities (EMA)*. 2021. https://www.nationalacademies.org/event/10-04-2021/docs/D18C8F5B242C6076541B8CC8F12932719C59AFDEC839
16. BioPhorum. *Peer to peer practical guidance on remote inspections and audits*. 2020. <https://www.biophorum.com/download/peer-to-peer-practical-guidance-on-remote-inspections-and-audits/>
17. Fujiwara Y. *PMDA pledge to tackle COVID-19 pandemic*. 2020. <https://www.pmda.go.jp/english/int-activities/0001.pdf>
18. Fujiwara Y. *PMDA Reveals Principles on Evaluation of COVID-19 Vaccines*. 2020. <https://www.pmda.go.jp/english/int-activities/0008.pdf>
19. Fujiwara Y. *PMDA Takes Further Steps to Speed up Clinical Development of COVID-19 Products*. 2020. <https://www.pmda.go.jp/english/int-activities/0002.pdf>
20. Fujiwara Y. *PMDA to Offer Free Scientific Advice for COVID-19 Vaccines Development*. 2020. <https://www.pmda.go.jp/english/int-activities/0007.pdf>
21. Pan American Health Organization (PAHO). *Regulatory System Strengthening in the Americas- Lessons Learned From the National Regulatory Authorities of Regional Reference*. 2021. <https://iris.paho.org/bitstream/handle/10665.2/53793/9789275123447_eng.pdf?sequence=5>
22. World Health Organisation (WHO) & International Coalition of Medicines Regulatory Authorities (ICMRA). *Report on the review of regulatory flexibilities/agilities as implemented by National Regulatory Authorities during Covid-19 pandemic*. 2021. <https://www.icmra.info/drupal/sites/default/files/2021-12/Regulatory_Flexibilities_during_COVID-19_Report.pdf>
23. Porrás A. *Pan American Network for Drug Regulatory Harmonization (PANDRH)* *Secretariat Report. 10th Conference: “The Regulatory Systems in the health agenda post COVID-19” PAHO/WHO Extraordinary virtual session* 2021. <https://www.paho.org/sites/default/files/x-cpandrh-secretariat-report-dec-2021-en_0.pdf>
24. International Conference of Drug Regulatory Authorities (ICDRA). *Plenary 5: Recommendations of Extraordinary Virtual ICDRA*. 2021. https://cdn.who.int/media/docs/default-source/medicines/regulatory-updates/virtual-icdra-2021/extra-icdra_plenary5_recommendations.pdf?sfvrsn=d7b1db92_5
25. Nakayama G. *APAC Thank you letter related to COVID-19*. 2021. https://apac-asia.com/images/achievements/pdf/10th/APAC%20thanks%20letter%20related%20to%20COVID-19.pdf
26. International Coalition of Medicines Regulatory Authorities (ICMRA). *ICMRA-Industry Virtual Workshop on Enabling Manufacturing Capacity in the COVID-19 Pandemic (Presentation)*. 2021. https://www.icmra.info/drupal/sites/default/files/2021-07/covid-19_manufacturing_capacity_ws_presentation.pdf

**Unpublished sources**:

1. Amgen. *Clinical Trial Regulatory Guidance During COVID-19 (ICMRA Health Authorities)*. 2021.
2. The International Federation of Pharmaceutical Manufacturers & Associations (IFPMA). *African Regulatory Network (ARN) Survey Results – Phase 1 (May 2020). Impact of COVID-19 on Regulatory activities in Africa*. 2020.
3. Drug Information Association (DIA). *Europe 2021 Outcome of the Industry Survey on Measures Put in Place in the EU During COVID-19 Crisis*. 2021.
4. Bernat J. IFPMA Letter to ICMRA - Clinical Trial agilities. 2021.
5. Bernat J. *Industry proposal recommendations: Manufacturing capacity for COVID-19 (ICMRA)*. 2021.
6. Association of the British Pharmaceutical Industry (ABPI). *Regulatory agilities during the COVID-19 pandemic and into the future*. 2021.
7. Beakes-Read G.(Amgen) *Regulatory flexibilities for clinical trials during the pandemic*. 2021
8. European Federation of Pharmaceutical Industries and Associations (EFPIA). COVID-19 Regulatory Flexibilities Survey; Lessons learned for Europe's future.
9. International Federation of Pharmaceutical Manufacturers & Associations (IFPMA). *COVID-19 – Regulatory Examples - September/October/November 2020 Updates*. 2020.
10. International Coalition of Medicines Regulatory Authorities (ICMRA) *Plenary 3: Industry Experience with Remote Assessments (Inspections) and Going Back to Normal*. 2021.

**Please note that any additional sources which were consulted during the development of the Review Article (not part of the literature review sources) are included in the References section of the Review Article itself.*
